# Rhynchophylline attenuates porcine pseudorabies virus-induced astrocyte injury by modulating oxidative stress, inflammation, and metabolic abnormalities

**DOI:** 10.3389/fphar.2026.1782776

**Published:** 2026-03-16

**Authors:** Xiaoyu Liu, Yalong Sun, Jiajia Wei, Chen Guan, Weiyi Yang, Jianqin Li, Shengjia Sun, Xue Zhang, Xianghua Shu, Huayi Bai, Ying Zhang, Deng Pan, Chunlian Song

**Affiliations:** 1 College of Veterinary Medicine of Yunnan Agricultural University, Kunming, Yunnan, China; 2 College of Animal Science and Technology of Yunnan Agricultural University, Kunming, Yunnan, China

**Keywords:** astrocytes, inflammation, metabolic abnormalities, oxidative stress, porcine pseudorabies virus, rhynchophylline, viral load

## Abstract

Porcine pseudorabies virus (PRV) causes astrocyte injury through oxidative stress, inflammatory responses, and metabolic dysfunction. Rhynchophylline (RHY) possesses antioxidant and anti-inflammatory properties, but its protective effects against PRV infection remain unclear. Using PRV-infected C8-D1A cells, we evaluated the antiviral and cytoprotective effects of RHY. At a non-toxic concentration of 5 μM, RHY significantly inhibited PRV replication, reduced intracellular reactive oxygen species, and alleviated oxidative stress by decreasing xanthine oxidase (XOD), myeloperoxidase (MPO), malondialdehyde (MDA), and nitric oxide (NO) levels while restoring superoxide dismutase (SOD) activity. RHY also modulated PRV-induced inflammatory imbalance by suppressing interleukin (IL)-6 and IL-8 and enhancing IL-4 and IL-10 expression. Metabolomic profiling revealed that PRV infection disrupted cellular metabolism, particularly pathways related to unsaturated fatty acid biosynthesis and the tricarboxylic acid (TCA) cycle, which were largely restored by RHY treatment. These findings indicate that RHY exerts antiviral, anti-inflammatory, and antioxidant effects by correcting PRV-induced metabolic disturbances *in vitro*.

## 1 Introduction

Porcine pseudorabies virus (PRV), a neurotropic alpha-herpesvirus, represents a significant threat to the global pig industry and can cause fatal encephalitis in pigs and other mammals (Freuling et al., 2017; Zheng et al., 2026). Astrocytes, which constitute the predominant glial cells within the central nervous system (CNS), are essential for preserving synaptic balance, modulating neurotransmitter metabolism, and facilitating neuronal nutrition (Sofroniew, 2020). Dysfunction in astrocytes can compromise CNS integrity and accelerate neurological disease progression (Zhuang et al., 2025). PRV infections in piglets and non-natural hosts often involve CNS invasion (Zhang Y. et al., 2025), triggering a cascade of pathological events such as oxidative stress (OS), inflammation, and metabolic reprogramming, which impair astrocyte function and disrupt CNS homeostasis (Zhou et al., 2023).

Following PRV infection, astrocytes become activated, releasing pro-inflammatory mediators that promote neuroinflammation, which exacerbates neuronal injury (Clark et al., 2021). The infection also leads to a disruption in the equilibrium of reactive oxygen species production and the antioxidant defense system, leading to excessive reactive oxygen species (ROS) buildup and lipid peroxidation (Wang et al., 2022), as indicated by elevated malondialdehyde (MDA) levels. Antioxidant enzyme activity, particularly superoxide dismutase (SOD), is reduced (Wang et al., 2022). Additionally, PRV activates the TLR-NF-κB signaling pathway, which triggers the secretion of pro-inflammatory cytokines including interleukin (IL)-6 and IL-8 while suppressing anti-inflammatory factors such as IL-4 and IL-10 (Zhou et al., 2023). The number of astrocytes increases post-infection, and their phenotype shifts from A2 to A1, reflecting heightened responsiveness to inflammatory signals (Zhang Y. et al., 2025). Recent studies suggest that metabolic reprogramming is another major consequence of PRV infection (Sasivimolphan et al., 2012). The virus exploits host metabolic pathways, encompassing glycolysis and the tricarboxylic acid (TCA) cycle, to meet its energy and biosynthetic needs, thereby disrupting normal cellular metabolism (Thai et al., 2014; Gualdoni et al., 2018). These interconnected pathological processes exacerbate PRV-induced astrocyte injury and CNS dysfunction.

Rhynchophylline (RHY), the primary active indole alkaloid extracted from the traditional Chinese medicinal herb *Uncaria rhynchophylla*, has gained attention for its potential in treating CNS disorders (Yang et al., 2020). RHY can scavenge ROS, limit the release of pro-inflammatory factors (Li et al., 2018), and modulate neuronal glucose metabolism (Wang et al., 2020). For instance, RHY alleviates OS and mitigates traumatic brain injury in rats (Zhong et al., 2025) while reducing neuroinflammation through regulation of cytokine balance (Li et al., 2021). However, despite these findings, the full spectrum of RHY’s protective effects against PRV-induced injury in C8-D1A astrocytes—such as inhibition of viral replication, preservation of cell viability, and restoration of metabolic homeostasis—remains underexplored.

To address this gap, this study established an *in vitro* model of PRV-infected C8-D1A astrocytes to evaluate RHY’s inhibitory effect on viral replication. Additionally, the study systematically analyzed RHY’s capacity to regulate post-infection inflammatory factor dysregulation and OS damage. Furthermore, metabolomic analysis using ultrahigh performance liquid chromatography-quadrupole time-of-flight mass spectrometry (UHPLC-Q-TOF/MS) was employed to scout how RHY modulates PRV-induced metabolic disturbances in C8-D1A astrocytes. This research aims to clarify the protective mechanism of RHY against PRV-induced damage in C8-D1A astrocytes and may provide novel therapeutic insights for managing PRV-associated CNS disorders.

## 2 Materials and methods

### 2.1 Cells, virus, and reagents

The mouse astrocyte cell line C8-D1A was procured from the American Type Culture Collection (ATCC, Manassas, VA, United States) and cultured in high-glucose DMEM supplemented with 10% FBS and 1% P/S (Gibco, Grand Island, NY, United States) at 37 °C in a 5% CO_2_ incubator.

The PRV-XJ strain [titer 1 × 10^6^ TCID_50_/mL] was provided by the Key Laboratory of Veterinary Pathobiology, Yunnan Agricultural University. RHY (purity ≥98%, HY-N0387) was purchased from Sigma-Aldrich (United States). The Cell counting kit 8 (CCK-8) assay kit was sourced from Beyotime Biotechnology (China), and the ROS detection kit (CellROX Green) from Thermo Fisher Scientific (United States). Assay kits for xanthine oxidase (XOD), SOD, myeloperoxidase (MPO), and nitric oxide (NO) were provided by Nanjing Jiancheng Bioengineering Institute (China). Total RNA extraction, cDNA synthesis, and SYBR Green qPCR Master Mix kits were obtained from Takara Bio (Japan). UHPLC-grade methanol and acetonitrile were supplied by Merck (Germany).

### 2.2 Experimental design

C8-D1A cells (2 × 10^5^ cells/well) were inoculated in 6-well plates, with each condition executed in triplicate and independently replicated three times. The cells were split into three treatment groups: the blank control group, which received normal DMEM; the PRV-infected group, where cells were infected with PRV (1 × 10^5^ TCID_50_/well) for 2 h, followed by replacement of the inoculum with fresh DMEM; and the RHY-treated group, where cells were pre-treated with the optimal concentration of RHY for 2 h, followed by PRV infection (1 × 10^5^ TCID_50_/well) for 2 h, after which the inoculum was replaced with fresh Dulbecco’s modified Eagle’s medium (DMEM) containing the optimal concentration of RHY. Cells and supernatants were collected at 12, 24, and 48 h post-infection for subsequent assays.

### 2.3 CCK-8 assay for optimal RHY concentration

C8-D1A cells (6 × 10^4^ cells/well) were plated in 96-well plates. After overnight incubation, various concentrations of RHY (0, 5, 10, 20, 40, 60, 80, 100, and 120 μM) were added, with six replicates per concentration. After 24 h of incubation, 10 μL of CCK-8 solution was introduced into each well, and incubation continued at 37 °C for two more hours. Absorbance was quantified at 450 nm with a microplate reader (Thermo Scientific, Waltham, MA, United States). Cell viability was computed as follows: $\text{Cell viability }\left(\%\right)==\frac{A_{s}-A_{b}}{A_{c}-A_{b}}\times 100.$

Where As is the absorbance of the sample well (containing cells, medium, the tested drug, and CCK-8 reagent), Ac is the absorbance of the control well (containing cells, medium, and CCK-8 reagent, without the drug), and Ab is the absorbance of the blank well (containing medium and CCK-8 reagent, without cells or drug).

### 2.4 Real-time quantitative polymerase chain reaction (RT-qPCR) for viral load and cytokine mRNA

#### 2.4.1 Viral load

Total RNA was extracted using TRIzol reagent (Vandesompele et al., 2002) and reverse-transcribed with the PrimeScript RT Reagent Kit. The PRV gE gene was amplified with certain primers (F: 5′-CGA​CAG​CGA​GCA​GAT​GAC​CA-3′, R: 5′-CTA​CAG​CGA​GAG​CGA​CAA​CGA-3′) and SYBR Green Master Mix on a StepOnePlus Real-Time PCR System (Thermo Fisher Scientific, United States). The cycling parameters were: 95 °C for 5 min, followed by 40 cycles of 95 °C for 30 s, 59.2 °C for 30 s, and 72 °C for 30 s (Bustin et al., 2009). The copy number of the PRV gE gene was determined using a standard curve (Y = −3.721X + 36.15, *R* ^2^ = 0.998), where X represents the logarithm of the PRV gE gene copy number and Y corresponds to the Ct value.

#### 2.4.2 Inflammatory factor mRNA

Primers for IL-4, IL-6, IL-8, IL-10, and the internal control β-actin are listed in Table 1. RT-qPCR was conducted under conditions identical to those used for viral load quantification. Relative expression was computed via the 2^−ΔΔCT^ method (Ruijter et al., 2009).

**TABLE 1:** The primer sequences of real-time quantitative PCR (RT-qPCR).

Gene name	Primer sequence
IL-4	F:5′-CCAAACGTCCTCACAGCAAC-3′
	R:5′-AGGCATCGAAAAGCCCCGAA-3′
IL-6	F:5′-TCTTGGGACTGATGCTGGTG-3′
	R:5′-GCCACTCCTTCTGTGACTCC-3′
IL-8	F:5′-ATTTCCACCGGCAATGAAGC-3′
	R:5′-GTCTCCCGAATTGGAAAGGGA-3′
IL-10	F:5′-GGCCCAGAAATCAAGGAGCA-3′
	R:5′-GCCTTGTAGACACCTTGGTCTT-3′
β-actin	F:5′-TGCTGTCCCCTGTATGCCTCTG-3′
	R:5′-TTGATGTCACGCACGATTTCC-3′

### 2.5 OS assays

#### 2.5.1 ROS detection

Following collection and two washes with PBS, cells were incubated with 5 μM CellROX Green reagent (Thermo Fisher Scientific, United States) at 37 °C for 30 min in the dark. After washing, ROS levels were quantified using a BD FACSCanto II flow cytometer (BD Biosciences, San Jose, CA, United States) with 488 nm excitation and 525 nm emission.

#### 2.5.2 Biochemical analysis of XOD, SOD, MPO, MDA, and NO

Following lysis in RIPA buffer containing 1% protease inhibitor (Beyotime) and centrifugation (12,000 × g, 15 min, 4 °C), the supernatant was gathered. Activities of SOD, XOD, MPO, MDA, and NO levels were gauged using commercial kits (Nanjing Jiancheng) according to the manufacturer’s protocols, with absorbance measured using a microplate reader.

### 2.6 Metabolomics analysis

#### 2.6.1 Sample preparation

Before being centrifuged (12,000 × g, 15 min, 4 °C), the cells were harvested, rinsed twice with ice-cold PBS, homogenized in a methanol–water (4:1, v/v) combination, and sonicated on ice for 10 min. After being dried under nitrogen, the supernatant was reconstituted in methanol–water (1:1, v/v), and passed through a 0.22 μm membrane. The samples were sent to Novogene Co., Ltd. (Beijing, China) for analysis.

#### 2.6.2 Ultrahigh performance liquid chromatography-quadrupole time-of-flight mass spectrometry (UHPLC-Q-TOF/MS) analysis

Chromatographic separation was achieved on an Agilent 1290 UHPLC system (Agilent Technologies, Santa Clara, CA, United States) fitted with an Agilent ZORBAX SB-C18 column (2.1 × 150 mm, 1.8 μm). The elution profile, using 0.1% formic acid in water (A) and acetonitrile (B) as mobile phases was: 0–2 min, 5% B; 2–10 min, 5%–95% B; 10–15 min, 95% B; 15–15.1 min, 95%–5% B; 15.1–20 min, 5% B. The system operated with 2 μL injection volume, a column temperature of 40 °C, and a flow rate of 0.3 mL/min.

An Agilent 6545 Q-TOF/MS system (Agilent Technologies) with electrospray ionization was implemented for detection in both ion modes (Zhou et al., 2017). The source settings were: capillary voltage, 3.5 kV (positive)/3.2 kV (negative); nozzle voltage, 500 V; drying gas (325 °C, 8 L/min); sheath gas (350 °C, 11 L/min). Data were gathered across the mass range of m/z 50–1,000.

#### 2.6.3 Data processing

Raw data were processed using Agilent MassHunter Qualitative Analysis (v B.07.00). Metabolites were identified by querying the Mouse Metabolome Database (MMDB) within the Human Metabolome Database (HMDB) (Wishart et al., 2022) and the METLIN database (with the species filter set to *Mus musculus*), matching exact mass (mass error <5 ppm), isotopic patterns, and MS/MS fragments. Differential metabolites were screened across time points, encompassing both the Ctrl versus PRV and the PRV versus RHY groups, using Partial Least Squares Discriminant Analysis (PLS-DA) and Orthogonal PLS-DA (OPLS-DA) in SIMCA-P 14.1 (Umetrics, Umeå, Sweden), with Variable Importance in Projection (VIP) >1 and *P* < 0.05 (Xia and Wishart, 2016). Enrichment analysis was implemented by the Kyoto Encyclopedia of Genes and Genomes (KEGG) (Kanehisa et al., 2023).

### 2.7 Statistical analysis

Data are presented as mean ± standard deviation (SD) from at least three independent experiments. Statistical analysis was performed with GraphPad Prism 10.0 (GraphPad Software, San Diego, CA, United States), using one-way ANOVA with Duncan’s multiple range test for group contrasts (Zhang, G. et al., 2025). Differences with *P*-value <0.05 and *P*-value <0.01 were represented considered statistically significant and highly significant, respectively.

## 3 Result

### 3.1 Viability of C8-D1A cells treated with RHY

**FIGURE 1:**
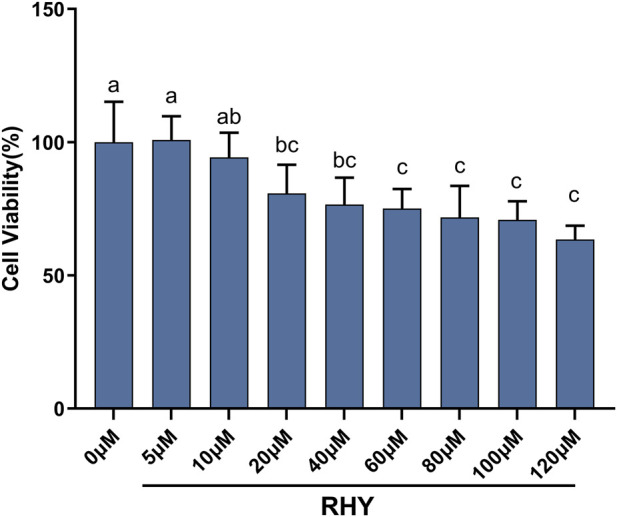
The effects of different concentrations of rhynchophylline (RHY) on the viability of C8-D1A cells were assessed using the cell counting Kit-8 (CCK-8) assay. Data are presented as mean ± standard deviation (SD) (n = 6). The same letters indicate no differences between groups (*P* > 0.05), while different letters represent significant differences between groups (*P* < 0.05).

The CCK-8 assay was utilized to assess the effects of various doses of RHY on C8-D1A cell viability (Figure 1). Within the same group, no substantial changes in cell viability were seen between the 5 μM and 10 μM RHY groups and the blank control (*P* > 0.05, cell viability ≥92%). However, a significant, concentration-dependent reduction in viability commenced at 20 μM (*P* < 0.05). Based on cytotoxicity and the requirements for subsequent experiments, 5 μM was chosen as the optimal RHY concentration.

### 3.2 Inhibition of PRV replication by RHY

**FIGURE 2:**
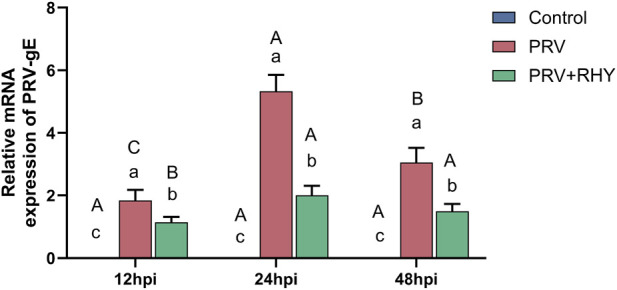
The effects of RHY on the expression levels of the PRV gE gene in C8-D1A cells were evaluated via real-time quantitative polymerase chain reaction (RT-qPCR) at 12, 24, and 48 h post-infection (hpi). Data are presented as mean ± SD (n = 3). Different lowercase letters (a, b, c) indicate significant differences between groups at the same time point (*P* < 0.05); different uppercase letters (A, B, C) indicate significant differences across time points within the same group (*P* < 0.05).

To gauge the inhibitory effect of RHY on PRV replication, the PRV gE gene’s expression was analyzed *via* RT-qPCR. The results demonstrated that at each time point, the viral load in the PRV-infected group was substantially heightened contrasted to the blank control, while the viral load in the 5 μM RHY treatment group was substantially lower than in the PRV-infected (*P* < 0.05) (Figure 2). In the PRV-infected group, the viral load peaked at 24 hpi and significantly decreased by 48 hpi (*P* < 0.05). In contrast, no significant variations were seen at different time points in the RHY treatment group (all labeled “b”, *P* > 0.05), confirming that RHY exhibited the most significant inhibitory effect during the peak of PRV replication at 24 hpi.

### 3.3 RHY restored inflammatory imbalance in PRV infection

**FIGURE 3:**
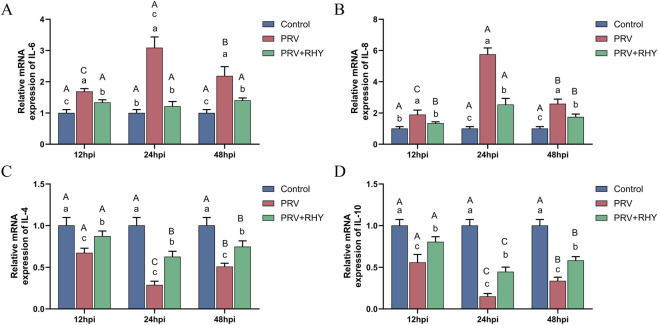
The effects of RHY on cytokine mRNA levels in C8-D1A cells were assessed. **(A–D)** The relative mRNA levels of interleukin (IL)-6 **(A)**, IL-8 **(B)**, IL-4 **(C)**, and IL-10 **(D)** were detected by RT-qPCR. Data are presented as mean ± SD (n = 3). Different lowercase letters (a, b, c) indicate significant differences between groups at the same time point (*P* < 0.05); different uppercase letters (A, B, C) indicate significant differences across time points within the same group (*P* < 0.05).

Given that PRV infection induces a robust systemic inflammatory response, the regulatory effect of RHY on the inflammatory imbalance triggered by PRV infection was assessed using RT-qPCR. The PRV-infected group’s IL-6 and IL-8 expression levels were markedly greater than those in the blank control at all time points (*P* < 0.05) (Figures 3A,B). In contrast, RHY treatment significantly reduced their expression compared to the PRV-infected group (*P* < 0.05). Within the PRV-infected group, IL-6 and IL-8 levels peaked at 24 hpi and significantly decreased by 48 hpi (all labeled “b”, *P* < 0.05), whereas no notable temporal variations were seen in the RHY-treated group. Conversely, relative to the blank control, the PRV-infected group exhibited markedly decreased expression levels of IL-4 (Figure 3C) and IL-10 (Figure 3D) (*P* < 0.05). RHY treatment significantly upregulated their expression relative to the PRV-infected group (*P* < 0.05). In the PRV-infected group, IL-4 and IL-10 levels were lowest at 24 hpi, while no notable variations were observed across time points in the RHY-treated group (all labeled “b”, *P* > 0.05). These findings unveil that RHY effectively restored the inflammatory imbalance induced by PRV infection.

### 3.4 RHY alleviated PRV-induced OS

**FIGURE 4:**
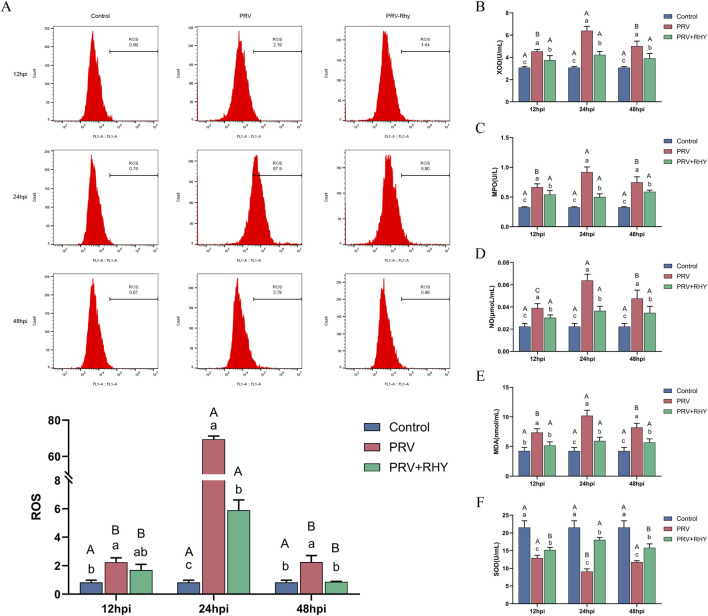
The effects of RHY on oxidative stress indicators in PRV-infected C8-D1A cells were investigated. **(A)** Reactive oxygen species (ROS) levels were detected by flow cytometry. **(B–F)** Xanthine oxidase (XOD) activity **(B)**, myeloperoxidase (MPO) activity **(C)**, nitric oxide (NO) content **(D)**, malondialdehyde (MDA) activity **(E)**, and superoxide dismutase (SOD) activity **(F)** were measured using biochemical assay kits. Data are presented as mean ± SD (n = 3). Different lowercase letters (a, b, c) indicate significant differences between groups at the same time point (*P* < 0.05); different uppercase letters (A, B, C) indicate significant differences across time points within the same group (*P* < 0.05).

To explore the potential of RHY in alleviating PRV-induced OS in C8-D1A cells, key OS markers were assessed. The levels of ROS (Figure 4A), XOD activity (Figure 4B), MPO activity (Figure 4C), NO content (Figure 4D), and MDA activity (Figure 4E) all followed similar trends. At each time point, these values in the PRV-infected group were substantially greater than those in the blank control and were markedly reduced by RHY treatment relative to the infected group (*P* < 0.05). In the PRV-infected group, these markers’ levels peaked at 24 hpi and significantly decreased by 48 hpi (*P* < 0.05). In contrast, no notable temporal changes were seen in the RHY-treated group. On the other hand, the activity of SOD (Figure 4F) in the PRV-infected group was dramatically decreased relative to the blank control (*P* < 0.05), but it was significantly restored by RHY treatment (*P* < 0.05). SOD activity in the PRV-infected group reached its lowest point at 24 hpi, while it remained stable in the RHY-treated group. These findings demonstrate that RHY efficiently alleviates PRV-induced OS by decreasing ROS, XOD, MPO, MDA, and NO levels, while restoring the activity of the antioxidant enzyme SOD.

### 3.5 RHY reversed PRV-induced metabolic disturbances in C8-D1A cells

**FIGURE 5:**
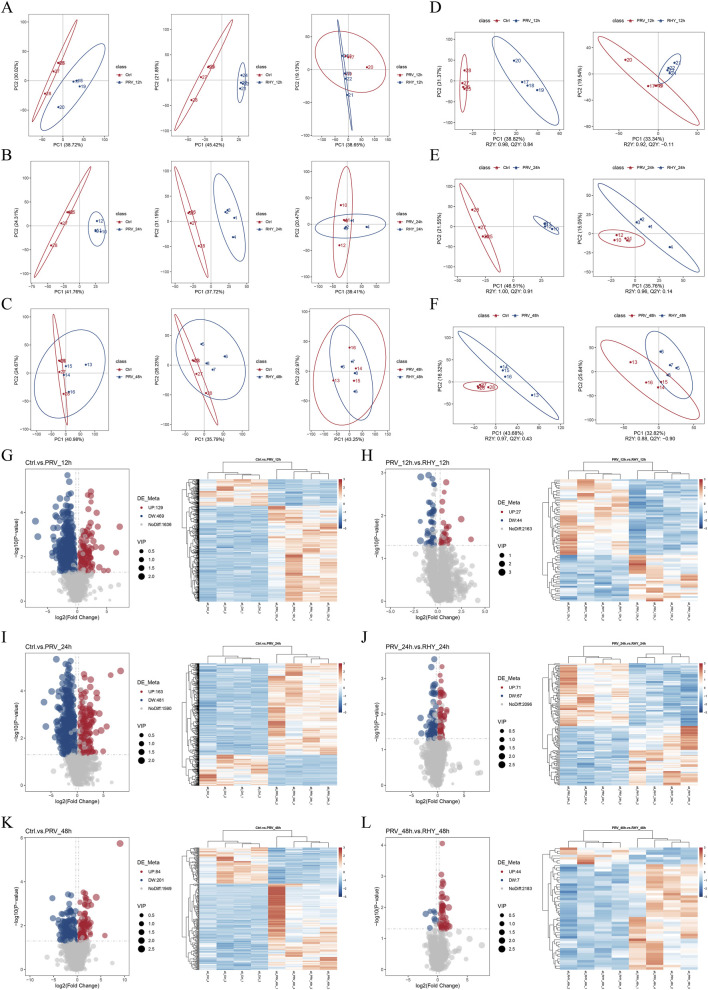
Metabolomic analysis of RHY’s effect on reversing PRV-induced metabolic dysregulation in C8-D1A cells was performed. **(A–C)** Principal component analysis (PCA) score plots of the control group (Ctrl), PRV-infected group, and RHY-treated group at 12 **(A)**, 24 **(B)**, and 48 **(C)** hours post-infection (hpi). **(D–F)** Partial Least Squares Discriminant Analysis (PLS-DA) score plots of the Ctrl, PRV, and RHY groups at 12 **(D)**, 24 **(E)**, and 48 **(F)** hours post-infection. **(G,H)** Volcano plots and heatmaps of differential metabolites between the Ctrl and PRV_12h groups **(G)**, as well as between PRV_12h and RHY_12h groups **(H)**. **(I,J)** Volcano plots and heatmaps of differential metabolites between the Ctrl and PRV_24h groups **(I)**, as well as between PRV_24h and RHY_24h groups **(J)**. **(K,L)** Volcano plots and heatmaps of differential metabolites between the Ctrl and PRV_48h groups **(K)**, as well as between PRV_48h and RHY_48h groups **(L)**.

To scout the regulatory effect of RHY on PRV-induced metabolic dysregulation in C8-D1A cells, UHPLC-Q-TOF/MS analysis was performed across various time points. Metabolic profiles of the blank control (Ctrl), PRV-infected, and RHY-treated groups were analyzed using principal component analysis (PCA) and PLS-DA. PCA results showed tight intra-group clustering, indicating high repeatability. A clear separation between the Ctrl and PRV groups was observed at 12 and 24 hpi along the PC1 axis, confirming that PRV infection significantly altered the cellular metabolic profile (Figures 5A,B). Notably, the RHY-treated group at 12 and 24 hpi clustered closer to the PRV group, suggesting that RHY could reverse infection-induced metabolic disturbances. However, at 48 hpi, there was overlap between the Ctrl and PRV, along with between the PRV and RHY groups (Figure 5C). PLS-DA further validated these observations (Figures 5D–F). The Ctrl vs. PRV model demonstrated excellent fit and predictive ability across time points. The PRV vs. RHY model exhibited clear separation between the infection and treatment groups, with no notable separation between the RHY and Ctrl groups in the PLS-DA plot, confirming that RHY effectively restored the metabolic homeostasis disrupted by PRV. This aligned with the earlier findings on the protective effects of RHY in reducing viral load and alleviating OS.

In the differential metabolite analysis, 598 differential metabolites were identified from Ctrl vs. PRV_12h, 71 from PRV_12h vs. RHY_12h, 644 from Ctrl vs. PRV_24h, 138 from PRV_24h vs. RHY_24h, 285 from Ctrl vs. PRV_48h, and 51 from PRV_48h vs. RHY_48h (Figures 5G–L). Further analysis revealed significant changes in metabolite levels over time among different treatment groups. At 12 hpi, alterations were detected in four metabolites (5,10-Methenyltetrahydrofolic acid, nor-toralactone, L-Leucyl-L-Valine, and 1-Pyrenemethanol). By 24 hpi, the number of altered metabolites increased to 43, including arachidonic acid, D-Gluconic acid, and threonic acid. At 48 hpi, changes were observed in 14 metabolites (D-Erythro-dihydrosphingosine, vomicine, and S-Acetyl dihydroasparagusic acid). These results indicate that RHY treatment significantly reversed these abnormalities.

### 3.6 Pathway enrichment of differential metabolites

**FIGURE 6:**
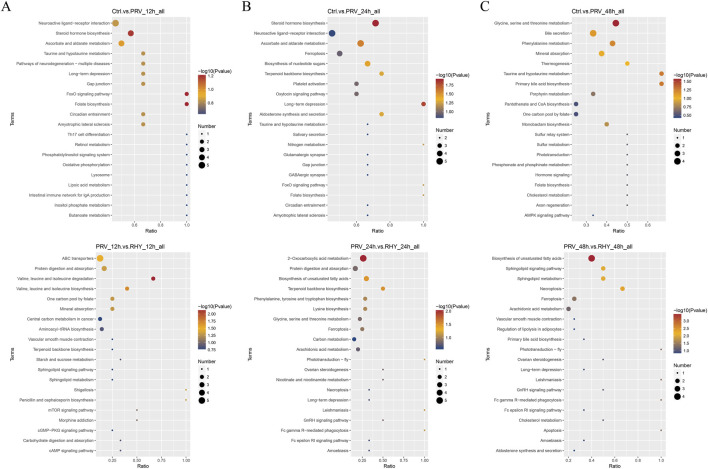
Kyoto Encyclopedia of Genes and Genomes (KEGG) enrichment analysis of differential metabolites across different groups. **(A)** Ctrl vs. PRV_12h groups and PRV_12h vs. RHY_12h groups. **(B)** Ctrl vs. PRV_24h groups and PRV_24h vs. RHY_24h groups. **(C)** Ctrl vs. PRV_48h groups and PRV_48h vs. RHY_48h groups.

To explore the functions of the differential metabolites across time points, KEGG analysis was performed. At 12 hpi, the differential metabolites between the Ctrl and PRV groups were primarily enriched in oxidative phosphorylation, Th17 cell differentiation, FoxO signaling pathway, and neuroactive ligand–receptor interaction. In contrast, the differential metabolites between the PRV and RHY groups showed significant enrichment in the cAMP and sphingolipid signaling pathways and aminoacyl-tRNA biosynthesis (Figure 6A). At 24 hpi, the differential metabolites between Ctrl and PRV groups were mainly enriched in steroid hormone biosynthesis, TCA cycle, and neuroactive ligand–receptor interaction. Additionally, the differential metabolites between the PRV and RHY groups were markedly enriched in unsaturated fatty acid biosynthesis, 2-oxy-carboxylic acid metabolism (TCA cycle-associated pathway), and glycine-serine-threonine metabolism (Figure 6B). By 48 hpi, differential metabolites between the PRV and RHY groups were linked to the AMPK signaling pathway, phosphonate and phosphinate metabolism, and glycine, serine, and threonine metabolism. Furthermore, differential metabolites between the PRV and RHY groups were primarily involved in Fc gamma R-mediated phagocytosis, sphingolipid signaling, and biosynthesis of unsaturated fatty acids (Figure 6C). PRV infection likely disrupts energy metabolism and CNS function by perturbing the TCA cycle and neuroactive signaling pathways, while RHY alleviates cellular damage by regulating lipid metabolism pathways.

## 4 Discussion

This study clearly demonstrated that RHY provides multi-dimensional protection against PRV-induced damage in C8-D1A astrocytes, acting through the inhibition of virus replication, reduction of OS, regulation of inflammatory levels, and reversal of metabolic abnormalities. These results not only enhance our comprehension of the antiviral potential of natural alkaloids but also furnish experimental support for the development of alternative strategies against PRV.

In line with the objective of evaluating RHY’s protective effect against PRV-induced astrocyte injury, the experimental design included three groups: blank control, PRV-infected, and RHY-treated groups. This design allowed for a clear differentiation of the independent effects of PRV infection and RHY treatment. The CCK-8 assay identified a non-cytotoxic concentration of 5 μM RHY as the optimal dose, laying the foundation for subsequent efficacy assessments. The time points of 12, 24, and 48 hpi were selected based on established alphaherpesvirus replication dynamics (Esteves et al., 2022), ensuring the analysis encompassed key stages of the infection cycle, including the expected replication peak at 24 hpi.

Early genes play a critical role in the production of progeny virus following PRV infection. Regarding virus replication inhibition, RT-qPCR results unveiled that RHY significantly reduced the copy number of the PRV gE gene at all time points, with the strongest inhibition occurring at 24 hpi, where a 68.3% reduction was observed. This time-dependent pattern is consistent with the known replication behavior of PRV in astrocyte-derived cells, where PRV completes early gene transcription and late protein assembly within 24 hpi. RHY’s efficiency is comparable to other natural products reported in the literature, such as berbamine, which reduces PRV load by 62.1% in PK-15 cells (Li et al., 2025), and resveratrol, which inhibits PRV replication by 59.3% in microglial cells (Luan et al., 2025). While RHY’s precise antiviral mechanism requires further investigation, it likely interferes with key steps in the viral life cycle, such as early gene transcription or late protein assembly. Future experiments should focus on viral protein expression and subcellular localization.

OS arises when there is an imbalance between oxidative and antioxidant systems. PRV induces high levels of OS in both *in vitro* and *in vivo* models (Sun et al., 2023), and antioxidants that restore redox balance can inhibit viral replication (Xu et al., 2025). The results of this study further support these findings: PRV infection in C8-D1A cells elevated ROS, XOD, MPO, MDA, and NO levels while decreasing SOD activity, consistent with prior reports that PRV activates NADPH oxidase and inhibits the antioxidant enzyme system. Additionally, astrocyte-derived mediators can influence microglial responses to Japanese encephalitis virus infection by promoting inflammation and OS (Mohapatra et al., 2023). RHY treatment effectively reversed these changes, consistent with its known antioxidant role in other disease models (Fu et al., 2021). For example, in a rat model of brain ischemia-reperfusion injury, RHY reduced ROS production by 45.2%, confirming its ability to restore intracellular redox balance. RHY has also been shown to effectively alleviate asthma inflammation by inhibiting the JAK2/STAT3 signaling pathway, reducing OS, and inhibiting autophagy-related proteins.

Cytokine storm is closely associated with PRV-induced pathological changes in tissues. In terms of inflammation regulation, PRV infection caused a significant cytokine imbalance, with increased pro-inflammatory factors (IL-6, IL-8) and decreased anti-inflammatory factors (IL-4, IL-10). The TLR-NF-κB signaling pathway is responsible for this typical inflammatory response. RHY treatment restored this balance by suppressing pro-inflammatory factors and promoting anti-inflammatory factors, bringing cytokine levels closer to normal. This immunomodulatory effect aligns with reports that RHY regulates the Th1/Th2 cytokine balance in autoimmune diseases, possibly through suppression of the NF-κB pathway (Wang et al., 2023) or stimulation of the STAT3/IL-10 anti-inflammatory axis (Murray, 2007). RHY exhibits anti-inflammatory, anti-arrhythmic, antihypertensive, and neuroprotective effects. It alleviates inflammation both *in vivo* and *in vitro* by blocking the SMAD and MAPK signaling pathways (Wang et al., 2019). Furthermore, RHY reduces amyloid plaque burden and decreases inflammation by modulating the ubiquitin-proteasome system, angiogenesis, and microglial function. In a 1-methyl-4-phenyl-1,2,3,6-tetrahydropyridine (MPTP)-induced mouse model, RHY demonstrated neuroprotective effects by reducing dopaminergic neuronal loss and reversing the secretion of inflammatory cytokines. In summary, the relevant conclusions of this study still need to be further verified by Western blotting or luciferase reporter assays. Moreover, future research can further explore the RHY on more extensive immune responses such as the type I interferon pathway, in order to improve the molecular mechanism research of its anti-PRV infection.

Non-targeted metabolomics technology was used to reveal the pathogenic mechanisms of PRV and the protective effects of RHY at the systemic metabolic level. Metabolomic analysis showed that PRV infection initially induced oxidative phosphorylation, gradually shifting to the TCA cycle and amino acid metabolism. This pattern is consistent with the known mechanism by which viruses hijack host metabolism to support their replication. Viral replication within host cells is highly dependent on host metabolites and energy supply to drive the synthesis of viral macromolecules and viral particle assembly (O’Neill et al., 2016). RHY treatment reversed the alterations in most metabolites, particularly at 24 hpi. Enrichment analysis further revealed that the differential metabolites between the PRV-infected and RHY-treated groups were markedly enriched in unsaturated fatty acid biosynthesis and related lipid metabolism pathways. This finding is consistent with previously reported metabolic regulatory effects of RHY. For example, one study demonstrated that RHY improved serum glucose and lipid abnormalities in an obese rat model (Bai et al., 2025). A separate research shown that RHY mitigated hepatic steatosis, decreased lipid accumulation, enhanced energy metabolism, and optimized glucose metabolism (Liu et al., 2023). It is hypothesized that RHY may alleviate the metabolic imbalance induced by PRV infection by modulating host lipid metabolism homeostasis.

In summary, the findings align with the study’s objectives and design, confirming that RHY protects C8-D1A astrocytes from PRV-induced injury through a concerted mechanism (Zhang et al., 2023). Evidence from viral replication, OS, inflammation, and metabolism elucidates RHY’s protective effects, providing strong support for its potential in PRV prevention and control (Yang et al., 2020; Zhang et al., 2023). Nonetheless, there are several limitations that warrant consideration in future study. First, as the experiments were conducted solely in C8-D1A cells, the present study is limited by species differences, and further evaluation of RHY’s protective efficacy against PRV infection in animal models (e.g., PRV-infected mice or pigs) is necessary before its clinical potential can be assessed. Second, the mechanistic exploration in this study was focused on a single concentration. Future work will need to systematically establish concentration gradients, determine the IC_50_ value of RHY against PRV, and conduct more in-depth dose-dependent mechanistic studies on this basis. Third, while the current metabolomics analysis focuses on central carbon metabolism, PRV-induced abnormalities in lipid metabolism, nucleotide metabolism, and other metabolic pathways, as well as the regulatory effects of RHY, targeted metabolomics detection is needed to comprehensively clarify the metabolic regulatory network of RHY. Fourth, this study cannot completely exclude the impact of RHY’s direct pharmacological effects on the experimental results, and subsequent research will adopt strategies including time-difference experiments of viral infection and drug treatment (e.g., RHY pre-treatment followed by PRV infection) and gene knockdown/overexpression targeting key viral replication genes to independently evaluate RHY’s direct regulatory effects on host cell oxidative stress and inflammation under conditions of clear viral replication inhibition, thereby enabling a more rigorous analysis of its dual mode of action. Finally, this study did not provide quantitative pharmacodynamic parameters (e.g., median cytotoxic concentration [CC50], median inhibitory concentration [IC50], selectivity index [SI]) as routine drug screening and comprehensive dose-effect relationship characterization were not performed, representing a limitation. These parameters will be supplemented via complete dose-effect curve analysis in subsequent studies.

## 5 Conclusion

This study demonstrates that RHY offers multi-dimensional protection against PRV-induced damage in C8-D1A astrocytes. The 5 μM RHY treatment effectively inhibited PRV replication and modulated OS and inflammatory imbalance induced by PRV. Metabolomic analysis further revealed that RHY significantly reversed PRV-induced metabolic disturbances. These findings provide experimental evidence for the antiviral potential of natural alkaloids and lay a foundation for developing RHY as a candidate agent for PRV control in veterinary practice.

## Data Availability

The datasets presented in this study can be found in online repositories. The names of the repository/repositories and accession number(s) can be found in the article/supplementary material.
